# Fast and effective pseudo transfer entropy for bivariate data-driven causal inference

**DOI:** 10.1038/s41598-021-87818-3

**Published:** 2021-04-19

**Authors:** Riccardo Silini, Cristina Masoller

**Affiliations:** Departament de Física, Universitat Politècnica de Catalunya, Rambla St. Nebridi 22, 08222 Terrassa, Spain

**Keywords:** Information theory and computation, Statistical physics, thermodynamics and nonlinear dynamics

## Abstract

Identifying, from time series analysis, reliable indicators of causal relationships is essential for many disciplines. Main challenges are distinguishing correlation from causality and discriminating between direct and indirect interactions. Over the years many methods for data-driven causal inference have been proposed; however, their success largely depends on the characteristics of the system under investigation. Often, their data requirements, computational cost or number of parameters limit their applicability. Here we propose a computationally efficient measure for causality testing, which we refer to as *pseudo transfer entropy* (pTE), that we derive from the standard definition of transfer entropy (TE) by using a Gaussian approximation. We demonstrate the power of the pTE measure on simulated and on real-world data. In all cases we find that pTE returns results that are very similar to those returned by Granger causality (GC). Importantly, for short time series, pTE combined with time-shifted (T-S) surrogates for significance testing strongly reduces the computational cost with respect to the widely used iterative amplitude adjusted Fourier transform (IAAFT) surrogate testing. For example, for time series of 100 data points, pTE and T-S reduce the computational time by $82\%$ with respect to GC and IAAFT. We also show that pTE is robust against observational noise. Therefore, we argue that the causal inference approach proposed here will be extremely valuable when causality networks need to be inferred from the analysis of a large number of short time series.

Unveiling and quantifying the strength of interactions from the analysis of observed data is a problem of capital importance for real-world complex systems. Typically, the details of the system are not known, but only observed time series are available, often short and noisy. A first attempt to try to quantify causality from observations was done 1956 by Wiener^1^ and formalized in 1969 by Granger^2^. According to Wiener-Granger causality (GC), given two processes *X* and *Y*, it is said that “*Y* G-causes *X*” if the information about the past of *Y* improves, in conjunction with the past of *X*, the prediction of the future of *X*, than the latter’s past alone. Since then, several variations have been proposed^3–8^, and have been applied to a broad variety of fields, such as econometrics^9–11^, neurosciences^12^, physiology^13^ and Earth sciences^14–18^ to cite a few.

An information-theoretic measure, known as Transfer Entropy (TE), a form of conditional mutual information (CMI)^19^, which approaches this problem from another point of view: instead of predicting the future of *X*, it tests whether the information about the past of *Y* is able to reduce the uncertainty on the future of *X*. Since its introduction by Schreiber^20^ in 2000, TE has found applications in different fields such as neurosciences^21–26^, physiology^27–29^, climatology^30,31^, finantial^32^ and social sciences^33^.

For Gaussian processes, for which the mutual information (MI) is known from the early years of information theory and its introduction in nonlinear dynamics^34^ is known for about 30 years, the equivalence between GC and TE is well established^35^. There are no clear links though between GC and TE for non Gaussian processes. In practical terms, while TE provides a model-free approach, the need of estimating several probability distributions makes TE substantially more computationally demanding than GC.

The success of the GC and TE approaches strongly depends on the characteristics of the system under study (its dimensionality, the strength of the coupling, the length and the temporal resolution of the data, the level of noise contamination, etc.). Both approaches can fail in distinguishing genuine causal interactions from correlations that arise due to similar governing equations, or correlations that are induced by the presence of common external forcings. In addition, when the system under study is composed by more than two interacting processes, GC and TE can return fake causalities, i.e., fail to discriminate between direct and indirect causal interactions. Many methods have been proposed to address these problems^36–49^; however, their performance depends on the characteristics of the data, and their data requirements, computational cost, and number of parameters that need to be estimated may limit their applicability.

The aim of this work is to propose a new, fast and effective approach for detecting causal interactions between two processes, *X* and *Y*. Our approach is based on the TE idea of uncertainty reduction: starting from the original TE definition^20^, by applying Gaussian approximations we obtain a simplified expression, which we refer to as *pseudo transer entropy* (pTE). When *X* and *Y* are Gaussian processes, we show that pTE detects, as expected, the same causal interactions as TE, which are, in turn, as those inferred by GC. However, we find that when *X* and *Y* are non-Gaussian, pTE also returns results that are fully consistent with those returned by GC. Importantly, for short time series, pTE strongly reduces the computational cost with respect to GC.

The code, freely available in *GitHub*^50^, has been built to provide a new, user-friendly and low-computational-cost tool that quickly returns, from a set of short time series, a inferred causal network. This will allow inter-disciplinary network scientists to find interesting properties of the system under study, without requiring any knowledge of the underlying physics. For experts in specific fields, the algorithm developed can be used as a first step to quickly understand which variables may play important roles in a given high-dimensional complex system. Then, as a second step, more precise methods, which are data and computationally more demanding, can be used to further understand the interactions between the variables that compose the backbone of the system, that was inferred by using the pTE approach.

This paper is organized as follows. In the main text we first consider synthetic time series generated with three stochastic data generating processes (DGPs) where the underlying causality is known: a linear system, a nonlinear system, and the chaotic Lorenz system (section *Models* presents the details of the three DGPs). We compare the performance of pTE, GC and TE in terms of the power and size, which are the percentage of times that a causality is detected when there is causality (power) and when there is no causality (size, also known as false discovery). Clearly, for a method to be effective, it must have a high power and a low size. Using the selected DGPs we demonstrate that pTE obtains similar power and size as GC while, for short time series, it allows a large reduction of the computational cost. Then, we demonstrate the suitability of pTE for the analysis of real world time series by considering two well-known climatic indices: the NINO3.4 and All India Rainfall (AIR). In the section *Additional Information* we present results obtained with several other DGPs, and we also compare our results with previous results reported in the literature. In the section *Methods* we present the derivation of the pTE expression and we also describe the statistical tests performed for determining the significance of the pTE, GC and TE values. In *Methods* we also present the implementation of the algorithms.

## Results

First, we use the three DGPs described in *Models* to compare the performance of pTE, GC and TE in terms of the power and size. If by construction there is no causality from *X* to *Y*, the percentage of times the causality is higher than the significance threshold returned by the surrogate analysis will be called “size” of the test, i.e., is the probability that a causality is detected when there is no causality by construction. On the other hand, if by construction *X* causes *Y*, the percentage of times the method finds causality from *X* to *Y* is called “power” of the test. With the surrogate analysis adopted, the causality between the original data will be compared to the maximum one found within 19 surrogates^51^, and the probability that the original data displays by chance the highest causality is $5\%$.

**Figure 1:**
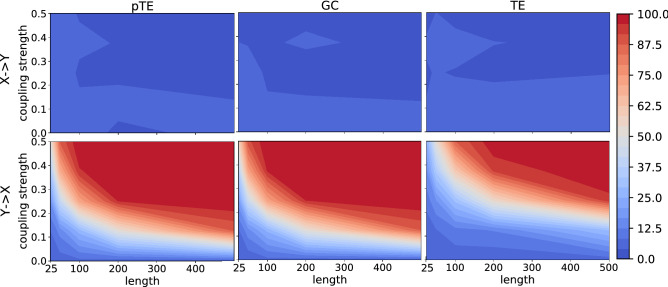
Power and size [the percentage of times that causality is detected when there is causality (power) and when there is no causality (size)] obtained using pTE (first column), GC (second column) and TE (third column) on the linear model, as a function of the length of the time series and of the strength of the coupling, for the two possible causality directions (top row: $X \rightarrow Y$, bottom row: $Y \rightarrow X$). By construction the model has causality from *Y* to *X*; therefore, the top row displays the size, and the bottom row, the power. The performance of pTE and GC is very similar, as both find the correct causality with moderate coupling strength even for short time series. TE finds the correct causality, but for stronger coupling.

**Figure 2:**
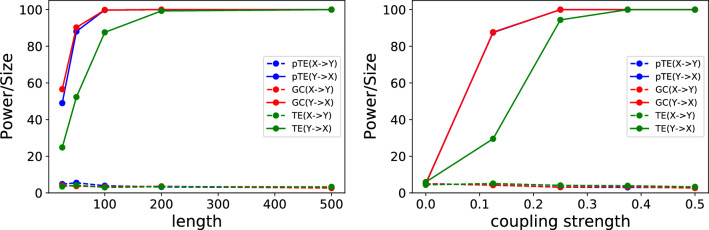
Power/size versus the coupling strength and the length of the time series (horizontal and vertical cross sections of Fig. 1). In the left panel, we fix the coupling strength to 0.5 and we plot the power/size of the linear model as a function of the time series length for pTE, GC and TE. In the right panel, we fix the number of data points to 500 and plot the power/size as a function of the coupling strength.

We analyze the power and size for the two possible causal directions ($X \rightarrow Y$ and $Y \rightarrow X$), as a function of the coupling strength and of the length of the time series. Figure 1 displays the power and size of the three methods, pTE, GC and TE, for the linear model, when the coupling is such that there is causality from *Y* to *X* (the size is shown in the top row, and the power, in the bottom row). The similarity between pTE and GC in finding the true causality is evident. With a coupling strength $C<0.1$ the three methods fail to detect causality, while for $C> 0.4$, for both pTE and GC, the number of data points in the time series needed to find causality is quite small, in fact 100 data points are sufficient to achieve a power of 100. In Fig. 2 we show results when we move along an horizontal or a vertical line in Fig. 1: we plot the power/size vs. the time series length, keeping fixed the coupling strength (left panel, $C=0.5$) and vs. the coupling strength, keeping fixed the time series length (right panel, $N=500$). In the left panel we notice that for $C=0.5$, a minimum of 200 data points are needed to retrieve the correct causality for all three methods with a power above 95. In the right panel, we notice that with 500 data points, a minimum coupling strength of $C\approx 0.25$ is necessary to find a power larger than 95 for all three methods.

**Figure 3:**
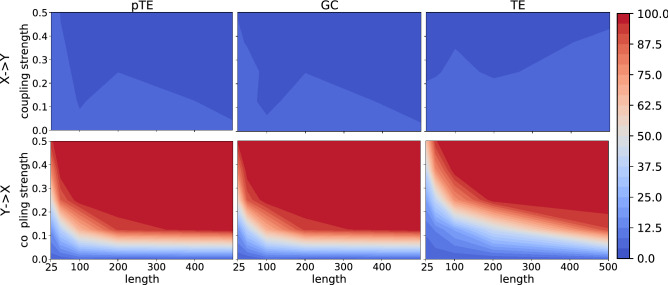
As Fig. 1, but using the nonlinear model. We again see that pTE and GC both find the correct causality, and their performance is very similar. TE finds the correct causality, but for stronger coupling.

**Figure 4:**
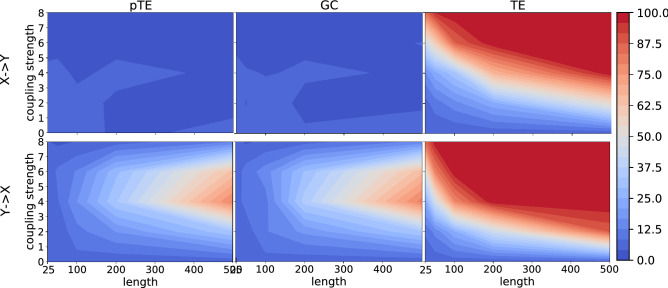
As Fig. 1, but using the chaotic model composed by two coupled Lorenz systems. The performance of pTE and GC is very similar, as both find the correct causality when the time series is long enough, and the coupling strength is moderate. TE finds $Y \rightarrow X$ causality, but it also finds $X \rightarrow Y$ causality, which is wrong by construction.

Figure 3 displays the results obtained for the nonlinear model, and we notice that they are very similar to the ones obtained with the linear model, probably due to the weak nonlinearity considered. We note that, in comparison with the linear model, in this model, with short time series the power and size returned by the three methods are more similar.

Regarding the two chaotic Lorenz oscillators, which are coupled in the first variable, the situation is very different, as shown in Fig. 4. When looking at the causality between the coupled variables, for both pTE and GC the causality is detected for a moderate coupling strength and a rather long time series. Causality $X \rightarrow Y$ is not detected for any (coupling strength, time series length), which is correct by construction. TE instead finds causality also for $X \rightarrow Y$, which is wrong by construction. This observation for TE can be attributed to insufficient conditioning treated by Paluš^19,52^, in fact the directionality of the coupling cannot be inferred when the systems are fully synchronized.

**Figure 5:**
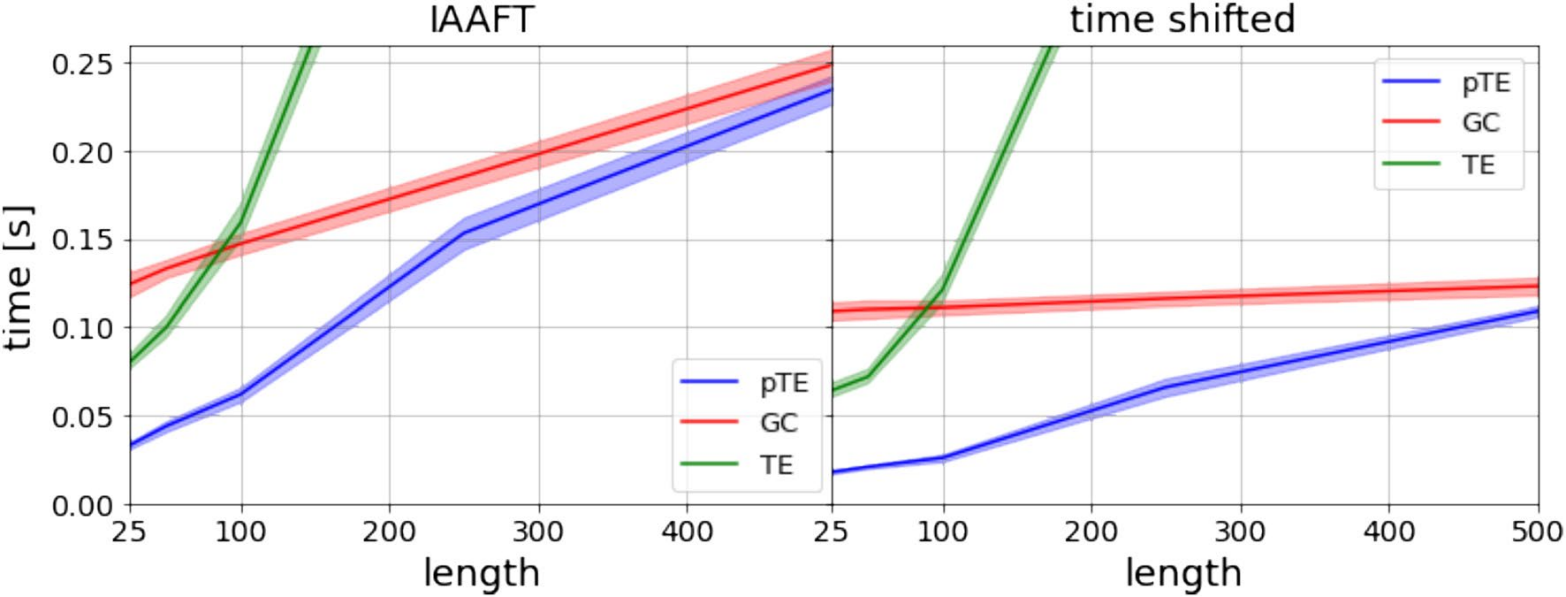
Computational times required to infer causal interactions in the two directions, $X \rightarrow Y$ and $Y \rightarrow X$, using pTE, GC or TE, as a function of the length of the time series, *N*. The times, calculated with the linear model after averaging over 1000 realizations, include pre-processing the time series and performing the statistical significance analysis. In the left panel IAAFT surrogates are used, while in the right panel time shifted surrogates are used.

Next, we compare the computational cost of using pTE, GC and TE. Figure 5 displays the time required to calculate $X \rightarrow Y$ and $Y \rightarrow X$ causalities, as a function of the length, *N*, of the time series. The figure shows the time required when the codes are run on Google colab CPUs ($\hbox {Intel}^{\tiny {\textregistered }}$ $\hbox {Xeon}^{\tiny {\textregistered }}$ CPU @ 2.20GHz), and includes preprocessing the time-series (detrending and normalizing) and performing the statistical significance test.

For short time series we see a large advantage of using pTE instead of GC. TE sits back as the slowest of the three methods. The reason is attributed to the scaling of parameter *k* in the *k*-nearest neighbors method used to compute TE, which scales as $\sqrt{N}$.

Table 1 displays the computational time required to calculate $X \rightarrow Y$ and $Y \rightarrow X$ causalities, and the corresponding power and size obtained using the linear model. While in Fig. 5 we showed the total computational time, in Table 1 we show only the time required for the calculation of the pTE, GC and TE values (without signal preprocessing and without performing statistical significance analysis). We see that, for time series of 25 data points, the time required for pTE calculation (averaged over 1000 runs) is 200% faster than GC; however, this percentage reduces to 12% for time series of 500 data points. From these results, we argue on the value of using pTE to analyze a large number of short time series, which is often the case when causality methods are used to build complex networks from observed data. We remark that all the codes used to generate the results shown in this article are publicly available at *GitHub*^50^.

**Table 1:** Average computational time of pTE, GC and TE per realization for four time series lengths, *N*. The mean and standard deviation are computed over 1000 realizations and the values in the table are expressed in milliseconds. pTE is the fastest up to $N=500$ data points, with the difference with GC diminishing as *N* increases. TE time increases exponentially as the *k* parameter of the *k*-nearest neighbors scales with $\sqrt{N}$. The power and size are computed for the linear model. We note that for $N=100$, pTE and GC give very similar results, even though pTE takes half the time. The last column displays the average computational cost reduction of pTE with respect to GC.

Data points N	Time (ms)			Power/size			Computational time reduction (%)
	pTE	GC	TE	pTE	GC	TE	
25	1.3 [[pm]] 0.3	3.7 [[pm]] 0.6	3.0 [[pm]] 0.5	49.0/4.8	56.6/4.1	24.8/3.5	64.9
100	1.9 [[pm]] 0.4	4.0 [[pm]] 0.6	8.6 [[pm]] 0.8	99.8/3.9	99.8/3.3	87.6/3.0	52.5
250	3.0 [[pm]] 0.6	4.6 [[pm]] 0.8	34 [[pm]] 2	100/3.2	100/3.6	99.3/3.4	34.8
500	4.1 [[pm]] 0.3	4.6 [[pm]] 0.3	112 [[pm]] 2	100/2.9	100/2.6	100/3.3	10.9

**Table 2:** Average computational time to generate time shifted (T-S) and IAAFT surrogates, for four time series lengths, *N*. The mean and standard deviation are computed over 1000 realizations and the values in the table are expressed in milliseconds. T-S surrogates are substantially faster than IAAFT, allowing to reduce the average computational time required to create surrogates by approximately $98\%$. The causality testing using pTE with the two surrogate methods gives very similar results in terms of power and size for the linear model.

Data points N	Time (ms)		Power/size		Computational time reduction (%)
	T-S	IAAFT	T-S	IAAFT	
25	0.020 [[pm]] 0.004	0.6 [[pm]] 0.1	48.9/3.2	51.5/4.9	96.7
100	0.035 [[pm]] 0.005	1.5 [[pm]] 0.3	97.5/0.0	99.2/3.0	97.7
250	0.07 [[pm]] 0.01	3.2 [[pm]] 0.6	100/0.0	100/2.9	97.8
500	0.13 [[pm]] 0.02	6 [[pm]] 1	100/0.0	100/2.6	97.8

The use of time-shifted (T-S) surrogates^51,53^ results in a substantial reduction of the computational time, in comparison to the widely used IAAFT surrogates, as seen in Fig. 5 and Table 2. The computational cost is reduced by approximately $98\%$, albeit displaying very similar results in terms of power and size. Clearly, T-S surrogates give a major boost in causality testing. As an example, for time series of length $N=100$, using pTE with T-S surrogates will reduce the computational cost by approximately $82\%$ with respect to GC with IAAFT surrogates, while a reduction of approximately $77\%$ is found with respect to GC with T-S surrogates. However, for causal inference T-S surrogates should be used with caution, because when there are time-delayed interactions, it can lead to fake conclusions.

**Figure 6:**
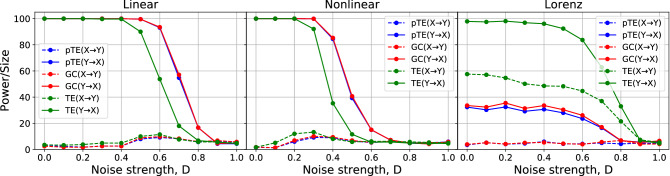
Resilience to noise of pTE, GC and TE, using the linear, nonlinear and chaotic models. pTE and GC perform very similarly (they are almost indistinguishable). The three measures are quite resilient to noise: for the linear model, up to 40% of noise can be present without significantly affecting the results, while for the nonlinear model, the three methods start failing at a lower noise strength. For the chaotic model, as previously noticed in Fig. 4, TE detects causality in both directions. The length of the time series is $N=300$ and the coupling strength is $C=0.5$ for the linear and nonlinear models, $C=4$ for the chaotic model (for this value of the coupling TE has the largest difference between the two directions).

To study the resilience to observational noise, we add, to the time series generated with the DGPs, *X* and *Y*, a Gaussian noise $\xi _{1,2}$ of zero mean and unit variance, tuning its contribution with a parameter $D\in [0,1]$. In this way we generate and analyse the signals $X^{'}$ and $Y^{'}$ given by $X^{'}_t = (1-D)X_t + D\xi _{1t}$, $Y^{'}_t = (1-D)Y_t + D\xi _{2t}$.

Figure 6 shows that pTE and GC perform very similarly (they are almost indistinguishable) and are quite resilient to noise. For the linear DGP, up to 40% of noise contribution can be present without a significant effect on the results, while for the nonlinear DGP, the methods start failing for a lower noise level. For the chaotic DGP the three methods are very resilient to noise. As previously noticed in Fig. 4, TE detects causality in both directions.

**Figure 7:**
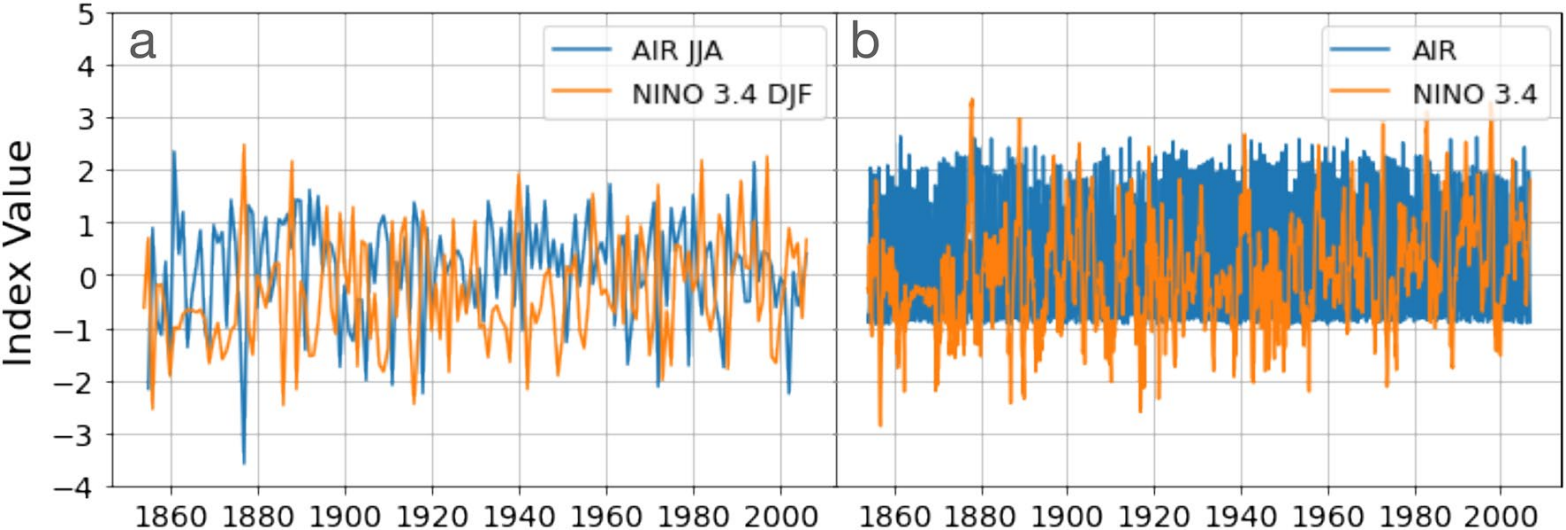
L2-normalized and linearly detrended time series of NINO3.4 and All India Rainfall (AIR) indices from 1854 to 2006. In panel (**a**) it is shown the average value of DJF for NINO3.4 index and JJA for AIR index, while in panel (**b**), the monthly sampled time series.

Finally, moving beyond synthetic data, we apply the pTE measure to two well-known climatic indices, and compare the results with GC and TE. The time series analysed, the NINO3.4 index and All India Rainfall (AIR) index, shown in Fig. 7, represent the dynamics of two large-scale climatic phenomena, the El Niño–Southern Oscillation (ENSO) and the Indian Summer Monsoon (ISM), whose causal inter-relationship is represented by long-range links (tele-connections) between the Central Pacific and the Indian Ocean^54^. The time series were downloaded from^55^. The NINO3.4 index begins in 1854 while AIR index begins in 1813. Monthly-mean values are available, and their shared period is from 1854 to 2006 (153 years, 1836 months),

Table 3 displays the results of the analysis of monthly-sampled data, and of yearly-sampled data. In the latter case we used the average of December, January and February (DJF) values, where the ENSO phenomenon peaks, and the average of June, July and August (JJA), where the monsoon peaks. Therefore, the length of the yearly-sample time series is 152 data points because for the last year the last data point, DJF, is not available. We used, for the yearly-sampled data, an autoregressive integrated moving average (ARIMA) model of order 4 (consistent with^16^) and, for the monthly-sampled data, of order 3. The order of the model was selected by using the Akaike information criterion (AIC).

**Table 3:** Results of the analysis of the NINO3.4 and AIR indices yearly- and monthly-sampled using T-S and IAAFT surrogates. The table indicates the the number of datapoints in the time series, the pTE, GC and TE values obtained, the significance threshold, and the computational time required to calculate the causality including statistical significance analysis.

Direction	N	pTE/th/sig.	Time(s)	GC/th/sig.	Time(s)	TE/th / sig.	Time(s)
**Time shifted**							
ENSO$\rightarrow$AIR	152	0.028/0.020/YES	0.02	1.9/1.8/YES	0.18	0.045/0.018/YES	0.35
AIR$\rightarrow$ENSO	152	0.003/0.029/NO	0.02	0.49/1.8/NO	0.18	0.06/0.02/YES	0.35
ENSO$\rightarrow$AIR	1836	0.002/0.006/NO	0.18	1.6/8.8/NO	0.38	0.03/0.02/YES	34
AIR$\rightarrow$ENSO	1836	0.0059/0.0058/YES	0.18	5.4/5.2/YES	0.38	0.02/0.04/NO	34
**IAAFT**							
ENSO$\rightarrow$AIR	152	0.028/0.020/YES	0.02	1.9/1.7/YES	0.20	0.07/0.04/YES	0.51
AIR$\rightarrow$ENSO	152	0.003/0.029/NO	0.02	0.49/1.8/NO	0.20	0.06/0.02/YES	0.51
ENSO$\rightarrow$AIR	1836	0.002/0.006/NO	0.25	1.6/8.8/NO	0.44	0.03/0.02/YES	34
AIR$\rightarrow$ENSO	1836	0.0059/0.0058/YES	0.25	5.4/5.2/YES	0.44	0.02/0.04/YES	34

In Table 3 we see that for the yearly-sampled data, pTE and GC only detect the dominant causality (ENSO$\rightarrow$AIR), while TE detects both (in good agreement with^16^). We note similarities with the results presented in Fig. 4: while unidirectional causality is found with pTE and GC, TE causality is found in both directions. The computational times clearly show that pTE is faster than GC (and of course also faster than TE, which is the slowest method). In the monthly-sampled data we see an opposite direction of causality, a result that we interpret as due to different time scales in the mutual influence between ENSO and ISM: while ENSO effects on the Indian monsoon precipitations are pronounced on an annual time scale, the influence of the Indian monsoon on ENSO acts on a shorter, monthly time scale. To exclude the fact that this change in directionality is an artifact due to the different time series lengths, we analyzed the monthly-sampled time series using segments of 152 consecutive data points (which is the length of the annually-sampled data). In this case we did not find any significant causality, which suggests that the change in directionality when considering annually-sampled or monthly-sampled data is not an artifact but has a physical origin, that we interpret as due to different time scales in the mutual interaction and that 152 data points are not sufficient to find causality (in any direction) in the monthly-sampled data.

Finally, we note that the computational times shown in Table 3 are higher than those that can be estimated from Fig. 5. In Fig. 5 we see that, for 150 datapoints, the time required for the GC calculation with T-S surrogate analysis is about 0.11 s while in Table 3 we see that the time required for GC and T-S calculation (two directions) is 0.36 s. The difference is due to the fact that in Fig. 5 a model of order 1 was used, while in Table 3, for the yearly-sampled data, a model of order 4 is used. The computational time increases with the order of the model, especially for GC, because the algorithm used (statsmodels grangercausalitytest) computes causality for all model orders up to the chosen one. For the NINO3.4 and AIR indices we also analysed the effect of varying the order of the model (from 1 to 10) and found either the same significant causal directionality (with stronger or weaker values), or we did not find any significant causality.

## Discussion

We have proposed a new measure, *pseudo transfer entropy* (pTE), to infer causality in systems composed by two interacting processes. Using synthetic time series generated with processes where the underlying causality is known, and also, a real-world example of two well-known climatic indices, we have found a remarkable similarity between the results of pTE and Granger causality (GC), in terms of the power and size, and the robustness to noise, but pTE can be significantly faster, particularly for short time series. For example, for time series of 100 datapoints, while giving extremely similar results, pTE with time-shifted (T-S) surrogate testing reduces the computational time by approximatelly 92% with respect to GC with IAAFT surrogate testing, and by 48% with respect to GC with T-S surrogate testing (on Google colab CPU, the total computational time for pTE and T-S is 2.5 ms, while for GC and IAAFT is 32.5 ms, and for GC and T-S, 4.7 ms).

Since the computational cost is of capital importance for the analysis of large datasets, the causality testing methodology proposed here will be extremely valuable for the analysis of short and noisy time series whose probability distributions are approximately Gaussian. We remark that many real-world signals follow distributions that are nearly normal. Although we do not claim that our method can be applied to any pair of signals, the information presented in the *Additional information* supports the method’s generic applicability. The algorithms are freely downloadable from *GitHub*^50^.

## Methods

### Derivation of the pseudo Transfer Entropy (pTE)

Transfer entropy^20^ is a well-known measure that quantifies the directionality of information transfer between two processes. In the case of information transfer from process *Y* to *X*, it is defined as

1 $$\begin{aligned} TE = \sum _{i,j} p\left( i_{n+1}, i_n^{(k)}, j_n^{(l)}\right) \log \left[ \frac{p\left( i_{n+1}\mid i_n^{(k)}, j_n^{(l)}\right) }{p\left( i_{n+1}\mid i_n^{(k)}\right) }\right] , \end{aligned}$$

where $p(\cdot , \cdot , \cdot )$ and $p(\cdot | \cdot )$ are joint and conditional probability distributions that describe the processes, $i_{n+1}$ represents the state of process *X* at time step $n+1$, $i_n^{(k)}$ and $j_n^{(k)}$ are shorthand notations that represent the states of *X* and *Y* the previous *k* time steps, $i_n^{(k)}=\{ i_n, \dots , i_{n-k+1}\}$, $j_n^{(k)}=\{ j_n, \dots , j_{n-k+1}\}$. Equation (1) can be re-written as

2 $$\begin{aligned} T_{Y\rightarrow X} = \sum _{i,j} p\left( i_{n+1}, i_n^{(k)}, j_n^{(l)}\right) \left\{ \log \left[ p\left( i_{n+1}\mid i_n^{(k)}, j_n^{(l)}\right) \right] - \log \left[ p\left( i_{n+1}\mid i_n^{(k)}\right) \right] \right\} , \end{aligned}$$

which, by using the definition of conditional probabilities and entropies, can be re-written as

3 $$\begin{aligned} T_{Y\rightarrow X} = H\left( i_n^{(k)}, j_n^{(l)}\right) - H\left( i_{n+1}, i_n^{(k)}, j_{n}^{(l)}\right) + H\left( i_{n+1}, i_n^{(k)}\right) - H\left( i_n^{(k)}\right) . \end{aligned}$$

The computation of the TE with Eq. (1) is challenging because a good estimation of the probability distributions is often not available. Considering the processes *X* and *Y* to follow normal distributions i.e. $X \sim {\mathscr {N}}(x\mid \mu _x, \Sigma _x)$ and $Y \sim {\mathscr {N}}(y\mid \mu _y, \Sigma _y)$, the computation simplifies substantially, using in fact that the entropy of a *p*-variate normal variable *x*, is given by

4 $$\begin{aligned} H_p\left( x\right) = \int _{-\infty }^{+\infty }{\mathscr {N}}(x\mid \mu _x, \Sigma _x) \log \left[ {\mathscr {N}}\left( x\mid \mu _x, \Sigma _x\right) \right] dx = -{\mathbb {E}}\left[ \log \left( {\mathscr {N}}(x\mid \mu _x, \Sigma _x)\right) \right] . \end{aligned}$$

By definition of the multivariate Gaussian, we can rewrite Eq. (4) as

5 $$\begin{aligned} H_p\left( x\right) = -{\mathbb {E}}\left[ \log \left( (2\pi )^{-\frac{p}{2}}\mid \Sigma \mid ^{-\frac{1}{2}} e^{-\frac{1}{2}(x-\mu _x)^{T}\Sigma _x^{-1}(x-\mu _x)} \right) \right] , \end{aligned}$$

which, by the property of the logarithm of products becomes

6 $$\begin{aligned} H_p\left( x\right) = \frac{p}{2}\log (2\pi ) +\frac{1}{2}\log (\mid \Sigma _x\mid ) + \frac{1}{2}{\mathbb {E}}\left[ (x-\mu _x)^T\Sigma ^{-1}(x-\mu _x)\right] . \end{aligned}$$

By noticing that ${\mathbb {E}}\left[ (x-\mu _x)^T\Sigma _x^{-1}(x-\mu _x)\right] = tr(\Sigma _x^{-1}\Sigma _x) = p$, we obtain

7 $$\begin{aligned} H_p(x) = \frac{1}{2}\left( p+p\log (2\pi ) + \log |\Sigma _x|\right) , \end{aligned}$$

where $|\Sigma |$ is the determinant of the $p \times p$ positive definite covariance matrix. By substituting Eq. (7) in Eq. (3), we can estimate the Transfer Entropy as follows:

8 $$\begin{aligned} \begin{aligned} TE_{Y\rightarrow X}&= \frac{1}{2}\left[ k+l + (k+l)\log (2\pi ) + \log \left( \left| \Sigma \left( {\mathbf {I}}^{(k)}_n\oplus {\mathbf {J}}^{(l)}_n \right) \right| \right) \right] \\&\quad - \frac{1}{2}\left[ k+l+1+(k+l+1)\log (2\pi ) + \log \left( \left| \Sigma \left( {\mathbf {i}}_{n+1}\oplus {\mathbf {I}}^{(k)}_n\oplus {\mathbf {J}}^{(l)}_n \right) \right| \right) \right] \\&\quad + \frac{1}{2}\left[ k+1 + (k+1)\log (2\pi ) + \log \left( \left| \Sigma \left( {\mathbf {i}}_{n+1}\oplus {\mathbf {I}}^{(k)}_n\right) \right| \right) \right] \\&\quad -\frac{1}{2}\left[ k+ k \log (2\pi ) + \log \left( \left| \Sigma \left( {\mathbf {I}}^{(k)}_n\right) \right| \right) \right] ,\\ \end{aligned} \end{aligned}$$

which finally can be written as

9 $$\begin{aligned} TE_{Y\rightarrow X} = \frac{1}{2} \log \left( \frac{\left| \Sigma \left( {\mathbf {I}}^{(k)}_n\oplus {\mathbf {J}}^{(l)}_n\right) \right| \cdot \left| \Sigma \left( {\mathbf {i}}_{n+1}\oplus {\mathbf {I}}^{(k)}_n\right) \right| }{\left| \Sigma \left( {\mathbf {i}}_{n+1}\oplus {\mathbf {I}}^{(k)}_{n} \oplus {\mathbf {J}}^{(l)}_n\right) \right| \cdot \left| \Sigma \left( {\mathbf {I}}^{(k)}_n\right) \right| }\right) , \end{aligned}$$

where $\Sigma (A\oplus B)$ is the covariance of the concatenation of matrices *A* and *B*, ${\mathbf {i}}_{n+1}$ is the vector of the future values of *X*, ${\mathbf {I}}^{(k)}_n$ and ${\mathbf {J}}^{(l)}_n$ are the matrices containing the previous *k* and *l* values of processes *X* and *Y* respectively. Whenever *X* and *Y* are not Gaussian processes, we call the quantity in Eq. (9) *pseudo Transfer entropy (pTE)*. For Gaussian variables pTE coincides with the Transfer Entropy and is equivalent to Granger causality^35^. The Gaussian form for CMI/TE for causality inference was also previously used^56–59^.

### Statistical significance

We used surrogate data to test the significance of the pTE, TE and GC values. The number of surrogates needed depends on the characteristics of the data, the available computational resources and time limitations: given enough resources and time, one should use a large number of surrogates and select a confidence interval^19^; however, with limited time or computational resources, when the spread of surrogates data is not too large one can use an alternative strategy: analyze a small number of surrogates and, in the case of a one sided test, select as significance threshold the maximum or minimum value obtained with the surrogates. In this case, $M = K/\alpha -$1 surrogates should be generated, where *K* is a positive integer number and $\alpha$ is the probability of false rejection^51^. Therefore, a minimum of 19 surrogates ($K=1$) are required for a significance level of $95\%$.

We used the algorithm developed by Schreiber and Schmitz^60,61^ known as *iterative amplitude adjusted Fourier transform* (IAAFT), which preserves both, the amplitude distribution and the power spectrum (for details, see Lancaster et al.^51^ and references therein). The python routine to compute the IAAFT surrogates is contained in the NoLiTSA package^62^. We also tested the time-shifted (T-S) surrogates^51,53^, which consist in randomly choosing a time shift independently for each surrogate and then shifting the signal in time, wrapping its end to the beginning. These surrogates are very fast to generate and they fully preserve all the properties of the original signal. Both surrogates test the null hypothesis of two processes with arbitrary linear or nonlinear structure but without linear or nonlinear inter-dependencies.

### Implementation

To calculate pTE we developed an algorithm in *python* (available on *GitHub*^50^), while we used the *statsmodels* implementation of GC^63^ and the *pyunicorn* implementation of TE^64^. The code has been thought to be as user friendly as possible to be used to build networks. It takes as arguments all the time series of the studied system, the embedding parameter and the statistical significance test that the user decides to apply. As result it returns the matrix of pTE values computed from the original data, and the matrix of the maximum values obtained from the surrogates (i.e., the statistically significant thresholds).

In the analysis of synthetic data generated with the DGPs the causality measures were run over 1000 realizations with different initial conditions and noise seeds. For each realization the first 100 data points were discarded. For the computation of GC and pTE we chose a lag equal to 1, which implies considering the models as auto-regressive processes of order 1, AR(1), since by the considered models construction, the dependent variable is influenced by the previous step of the independent one; for the computation of TE the *k*-nearest neighbors method is used, and we chose $k=\sqrt{N}$, where *N* is the number of data points in the time series^65^.

In the analysis of the empirical data, from the physics of the problem, the choice of the order of the AR model used to represent the data is not trivial. We used an autoregressive integrated moving average (ARIMA) and the Akaike information criterion (AIC) to select order 4 for yearly-sampled data and order 3, for the monthly-sampled data.

To calculate the causality between two time series, the time series were first linearly detrended and L2-normalized. The significance of the pTE, GC and TE values obtained were then tested against the values obtained from 19 couples of surrogates (as explained in the previous section, 19 surrogates is the minimum for achieving a significance level of $95\%$). Unless otherwise specifically stated, the results presented in the text were obtained by using IAAFT surrogates.

## Models

In the main text three data generating processes (DGPs) were analyzed. For these DGPs the null hypothesis of non-causality is not satisfied for process *Y* to process *X*. Results obtained with other DGPs are presented in the *Additional information*.

The first DGP is a linear model^66^ given by:

10 $$\begin{aligned} X_t=0.6X_{t-1} + C\cdot Y_{t-1} +\epsilon _{1t}, \qquad Y_t = 0.6Y_{t-1} + \epsilon _{2t}, \end{aligned}$$

where $\epsilon _{1t}$ and $\epsilon _{2t}$ are white noises with zero mean and unit variance, and *C* is the coupling strength.

The second DGP is a nonlinear model^67^ that reads:

11 $$\begin{aligned} X_t = 0.5X_{t-1}+C\cdot Y_{t-1}^2 +\epsilon _{1t}, \qquad Y_t = 0.5Y_{t-1} + \epsilon _{2t}. \end{aligned}$$

The third DGP consists of two Lorenz chaotic systems, coupled on the first variable:

12 $$\begin{aligned} \begin{array}{ll} {\dot{X}}_{1} = 10(-X_1+X_2)+ C\cdot (Y_1-X_1) &{}\quad {\dot{Y}}_{1} = 10(-Y_1+Y_2)\\ {\dot{X}}_{2} = 21.5X_{1} - X_{2} -X_1X_3 &{}\quad {\dot{Y}}_{2} = 20.5Y_{1} - Y_{2} -Y_1Y_3 \\ {\dot{X}}_{3} = X_{1}X_2 - \frac{8}{3}Y_3 &{}\quad {\dot{Y}}_{3} = Y_{1}Y_2 - \frac{8}{3}Y_3\\ \end{array} \end{aligned}$$

**Figure 8:**
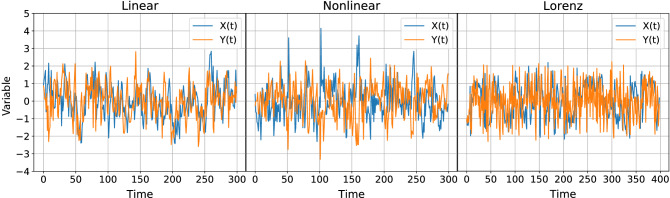
Examples of time series of the three data generating processes (DGPs) analyzed in the main text. In the three cases there is causality from *Y* to *X*; the coupling strength is (**a**), (**b**) $C=0.5$, (**c**) $C=8$.

Examples of time series of these three DGPs, normalized to zero mean and unit variance, are displayed in Fig. 8.

## Additional information

### Comparison with literature

The linear DGP was used by Diks and DeGoede^66^ to test nonlinear Granger causality. With a coupling strength of $C=0.5$ and a time series length of 100 points with a lag of 1, they obtained a power of 95.6 and a size of 3.0. Using pTE under the same conditions, we obtain a power of 99.8 and a size of 3.9.

The nonlinear DGP was used by Taamouti et al.^67^ to quantify linear and nonlinear Granger causalities. With a coupling strength of $C = 0.5$, 200 data points, a pvalue of 5% and a resampling bandwidth *k* for the bootstrap as the integer part of $2 \cdot 200^{1/2}$, they obtained a power of 100 and a size of 4.4. Using pTE we obtained a power of 100 and a size of 3.3.

The coupled Lorenz systems studied by Krakovská et al.^68^, are very similar to those studied here. By using three state-space based methods, including cross-mapping, they noticed that the highest directionality in the causality is for a coupling $C \approx 4$. From $C > 4$ synchronization is obtained, finding causality in both directions, using time series of 50000 data points. This observation is very similar to our results with TE, while for pTE and GC, once synchronization has been achieved, no causality is found. This supports their conclusion, warning the reader that the blind application of causality test can easily lead to incorrect conclusions. While GC and pTE can successfully be used to analyze AR processes and weakly nonlinear Gaussian-like processes, for more complex processes (high dimensional and/or highly nonlinear) advanced information-theoretic methods such as TE are needed.

### Additional data generating processes analyzed

**Table 4:** List of DGPs studied for the comparison between pTE, GC and TE (the results are reported in Table 5). Models M0–M2 have no causality by construction. Models M3–M11 have causality from *Y* to *X*, while M12–M14 have bidirectional causality. M0 is Gaussian white noise, M1 is a bivariate process with a linear dependence, M2 corresponds to spurious causality and M3 corresponds to a nonlinear model^67^. M4 is a nonlinear model where the *t*-th point of process *X* is built using the an autoregressive model of order 2, and it’s influenced by the $t-3$ value of process *Y*^69^. M5 is a heteroskedasticity mean causality, M6 a heteroskedasticity variance, while M7 is an homoskedasticity^70^. M8 and M9 have instantaneous causalities^71^, and M10 is a nonlinear ARX model^72^. M11 are two Rössler systems^73^ coupled by the first variable. M12 and M13 are the circle map^74^ with unidirectional and bidirectional causality respectively. M14 has bidirectional causality^67^.

Model	*X*	*Y*	Causality
M0	$\epsilon _{1t}$ white noise	$\epsilon _{2t}$ white noise	Y $\not \rightarrow$ X
M1	$x_t \sim {\mathscr {N}}(0,1,\gamma _{xy})$, $\gamma _{xy}=0.5$	$y_t \sim {\mathscr {N}}(0,1,\gamma _{xy})$, $\gamma _{xy}=0.5$	Y $\not \rightarrow$ X
M2	$x_t = (0.01 +0.5x_{t-1}^2)^{0.5}\epsilon _{1t}$	$y_t = 0.5y_{t-1} + \epsilon _{2t}$	Y $\not \rightarrow$ X
M3	$x_t = 0.5x_{t-1}y_{t-1}+ \epsilon _{1t}$	$y_t = 0.5y_{t-1} + \epsilon _{2t}$	Y $\rightarrow$ X
M4	$x_t=0.1+0.4x_{t-2} + \frac{2.4-0.9y_{t-3}}{1+e^{-4y_{t-3}}} +\epsilon _{1t}$	$y_t = 0.7y_{t-1} + \epsilon _{2t}$	Y $\rightarrow$ X
M5-M7	$x_t = 0.25x_{t-1}+ 0.5y_{t-1} + \sigma _{1t}$	$y_t = 0.2 + 0.1y_{t-1} + \sigma _{2t}$	Y $\rightarrow$ X
	$$\sigma _{it} = \eta _{it}\sqrt{H_{iit}},\quad \eta _{it} \sim {\mathscr {N}}(0,1)$$		
	$$H = \begin{pmatrix} 1 &{} 1\\ 1 &{} 1 \\ \end{pmatrix} + A\begin{pmatrix} \sigma _{1t} \\ \sigma _{2t} \\ \end{pmatrix}\begin{pmatrix} \sigma _{1t} \\ \sigma _{2t} \\ \end{pmatrix}^{T}A^T$$		
	$$M4: A = \begin{pmatrix}0.2 &{} 0.0 \\ 0.0 &{} 0.9\end{pmatrix} \quad M5: A = \begin{pmatrix}0.2 &{} 0.7 \\ 0.0 &{} 0.9\end{pmatrix} \quad M6: A = \begin{pmatrix}0.0 &{} 0.0 \\ 0.0 &{} 0.0\end{pmatrix}$$		
M8	$$\begin{aligned} x_t&= 0.65x_{t-1} + 0.38y_{t-1} + 0.01x_{t-2}\\&\quad - 0.21y_{t-2}+ \epsilon _{1t}\\ \end{aligned}$$	$$\begin{aligned} y_t&= 1.29y_{t-1} + 0.18x_{t-1} -0.35y_{t-2} \\&\quad -0.16x_{t-2}+ \epsilon _{2t} \end{aligned}$$	Y $\rightarrow$ X
M9	$$\begin{aligned} x_t&= 0.06x_{t-1} - 1.14y_{t-1} + 0.48x_{t-2}\\&\quad + 0.51y_{t-2} - 0.23x_{t-3} -0.51y_{t-3}+ \epsilon _{1t}\\ \end{aligned}$$	$$\begin{aligned} y_t&= 1.1y_{t-1} - 0.09x_{t-1} -0.36y_{t-2} \\&\quad -0.29x_{t-2}+ 0.09y_{t-3} - 0.15x_{t-3}\epsilon _{2t} \end{aligned}$$	Y $\rightarrow$ X
M10	$$\begin{aligned} x_t&=0.5x_{t-1}-0.3x_{t-2}+0.1y_{t-2}+0.1x_{t-2}^2+\\&\quad +0.4y_{t-1}y_{t-2}+\epsilon _{1t}\\ \end{aligned}$$	$y_t = \sin (4\pi t) +\sin (6\pi t) + \epsilon _{2t}$	Y $\rightarrow$ X
M11	$$\begin{aligned} {\dot{x}}_{1}&= -(1+0.015)x_{2} - x_{3} + 0.1 (y_{1} - x_{1})\\ {\dot{x}}_{2}&= (1+0.015)x_{1} + 0.15 x_{2}\\ {\dot{x}}_{3}&= 0.2 + x_{3}(x_{1} -10)\\ \end{aligned}$$	$$\begin{aligned} {\dot{y}}_{1}&= -(1-0.015)y_{2} - y_{3}\\ {\dot{y}}_{2}&= (1-0.015)y_{1} + 0.15 y_{2}\\ {\dot{y}}_{3}&= 0.2 + y_{3}(y_{1} -10)\\ \end{aligned}$$	Y $\rightarrow$ X
M12-M13	$$\begin{aligned} x_t&= \left( x_{t-1}+\rho + \frac{K}{2\pi } \sin (2\pi x_{t-1})\right. +\\&\quad + \beta \epsilon _{1t}\bigg ) \mod 1 + C_1 (x_{t-1}-y_{t-1})\end{aligned}$$	$$\begin{aligned} y_t&= \left( y_{t-1}+\rho + \frac{K}{2\pi } \sin (2\pi y_{t-1}) \right. \\&\quad + \beta \epsilon _{2t}\bigg ) \mod 1 + C_2 (y_{t-1}-x_{t-1})\end{aligned}$$	Y $\leftrightarrow$ X
	$$\rho =0.23, \quad K = 0.04,\quad \beta = 0.002$$		
	M11: $C_1 = 0.5, C_2=0$, M12: $C_1 = C_2= 0.5$		
M14	$x_t = 0.3 + 0.15x_{t-1}+0.7y_{t-1} + \epsilon _{1t}$	$y_t = 0.2 + 0.1y_{t-1}+0.8x_{t-1} + \epsilon _{2t}$	Y $\leftrightarrow$ X
	$$\begin{pmatrix} \epsilon _{1t} \\ \epsilon _{2t} \\ \end{pmatrix} \sim {\mathscr {N}}\left[ \begin{pmatrix} 0.0 \\ 0.0 \\ \end{pmatrix},\begin{pmatrix} 1 &{} 0.2 \\ 0.2 &{} 1 \\ \end{pmatrix}\right]$$

**Table 5:** Power and size obtained with the DGPs listed in Table 4 using pTE, GC and TE. We can notice that there are no significant differences between pTE and GC. The results were obtained using time series of length 1000, where the first 100 are discarded and they are averaged over 1000 realizations. The last three columns correspond to the directionality index DI, eg. $(pTE_{Y\rightarrow X} -pTE_{X\rightarrow Y})/(pTE_{Y\rightarrow X} + pTE_{X\rightarrow Y})$, which shows that pTE performs better in most of the models in assessing the directionality. The pTE has been calculated with an embedding parameter of 1 for all models except for M10, where an embedding parameter of 2 has been used to match the causality lag imposed by construction.

Model	pTE		GC		TE		DI		
	Y $\rightarrow$ X	X $\rightarrow$ Y	Y $\rightarrow$ X	X $\rightarrow$ Y	Y $\rightarrow$ X	X $\rightarrow$ Y	pTE	GC	TE
M0	**3.8**	**3.9**	5.1	5.0	4.4	4.4	$-0.01$	0.01	**0.00**
M1	**2.3**	**2.6**	3.3	3.1	*100*	*100*	$-0.06$	0.03	**0.00**
M2	**4.2**	**4.7**	5.5	5.9	4.7	4.9	$-0.06$	$-0.04$	$-{\mathbf{0.02}}$
M3	**100**	**4.5**	**100**	4.8	70.2	5.6	**0.91**	**0.91**	0.85
M4	80.7	**3.8**	84.2	4.9	**96.0**	4.7	**0.91**	0.89	**0.91**
M5	**100**	**2.2**	**100**	3.1	**100**	3.8	**0.96**	0.94	0.93
M6	**100**	**1.8**	**100**	2.8	**100**	4.3	**0.96**	0.95	0.92
M7	**100**	**2.8**	**100**	3.4	**100**	4.0	**0.95**	0.93	0.92
M8	**100**	**4.5**	**100**	5.6	**100**	*100*	**0.91**	0.89	*0.00*
M9	**100**	0.1	**100**	0.1	**100**	*100*	**1.00**	**1.00**	*0.00*
M10	62.6	**3.1**	**67.3**	4.3	12.2	4.5	**0.91**	0.88	0.46
M11	46.1	**43.1**	**53.1**	49.8	37.8	45.0	0.03	0.03	$-0.09$
M12	99.9	1.0	**100**	0.9	**100**	**0**	**1.0**	**1.0**	**1.0**
M13	**100**	**100**	**100**	**100**	**100**	**100**	**0.00**	**0.00**	**0.00**
M14	**100**	**100**	**100**	**100**	**100**	**100**	**0.00**	**0.00**	**0.00**

Bold indicates best values obtained for each model; italic indicates the wrong values obtained for each model.
